# COGNITOMICS: a new approach for the evaluation of the relationship between diseases and cognition to be designed based on normal behavioral tendencies in real life, a conceptual framework

**DOI:** 10.1186/s40200-017-0301-y

**Published:** 2017-05-15

**Authors:** Ata Pourabbasi, Bagher Larijani

**Affiliations:** 1 Diabetes Research Center, Endocrinology and Metabolism Clinical Sciences Institute, Tehran University of Medical Sciences, Tehran, Iran; 2 Endocrinology & Metabolism Research Center, Endocrinology and Metabolism Clinical Sciences Institute, Tehran University of Medical Sciences, Tehran, Iran; 3 Shari’ati Hospital, North Kargar St., Tehran, Iran

**Keywords:** Disease, Cognition, Behavior

## Abstract

The relationship between medical diseases and cognition has been a point of interest in the last decades. In recent years studies in this field have developed significantly applying various tools such as cognitive tests using psychometric assessments and brain imaging techniques. The main focus of cognitive function in this studies is the process through which this cognition is obtained. It seems a novel methodology is needed while assessing the impact of diseases such as diabetes on cognition based on heavenly religious teachings in which cognition is believed to be the main source of human bliss and not just some measurable quantitative components.

The relationship between medical diseases and cognition has been a point of interest in the last decades. The first marks on the impact of diseases on cognitive function dates back to the evaluation of consciousness in some chronic disease such as diabetes and liver disease about 60 years ago [1, 2].

But, in recent years studies in this field have developed significantly applying various tools such as cognitive tests using psychometric assessments and brain imaging techniques. All these studies show that components of cognition, including memory, attention and executive functions can be seriously affected by chronic diseases in patients of different ages [3, 4]. Later, the structural and functional studies of the nervous system also revealed structural changes in certain brain regions of people with chronic health condition, which can explain the observed changes in cognition [5].

Meanwhile, a question is raised that whether chronic health conditions affect the cognition of individuals as the most important factor affecting the behavior or solely affects a number of contractual components which have been accepted by some psychology schools and referred as cognitive areas? Another question is that if anatomical structures and neuronal physiological processes play a causative role in the cognitive capacity of people?

To answer these questions, one must pay greater attention to the meaning of cognition in the teachings of heavenly religions.

What defined as the cognition in the heavenly teachings is the ability of each individual in understanding the reality. Religious scholars maintain that the issue of cognition is such an obvious concept needing no illustration; however, is not so directly visible and evident [6].

The contextual cognition of the individual makes him able to come with an understanding of self and others, and to develop a knowledge-based worldview. Such a worldview contributes to achieving ideological do’s and not to do’s which forms the structure of behavioral norms.

Therefore, cognition in this perspective is a process which cannot be defined and observed all alone and is visible through the behavioral output [7].

This behavior, based on human cognitive system, could guarantee the human bliss. The important notion in this viewpoint is the behavior resulted by the cognitive ability, which is emphasized as the good deed by the heavenly teachings such as teachings of Islam, Judaism and christianity. This is the behavior that has originality and one should strive to maintain it as normative.

From this perspective, cognition may be acquired through an empirical pathway which means that sensory perception turns into cognition through a natural process. This path is not the only way through which a person can obtain cognition, but also cognition is obtained through an intuitive pathway [8].

From this point of view, behavior is considered as the most important and original conception which is the achievement of the cognition.

But, what considered as cognition in recent scientific movements is a set of capabilities such as attention, concentration, executive functions, problem solving, etc. which totally provides an understanding of self and others for the individual [9].

In other words, the main focus of cognitive function in this view is the process through which this cognition is obtained. However, behavior, as the only visible output of cognition, is not contemplated remarkably, and in the best case, is viewed the same as other cognitive components and no special place is given to it. On the same basis, direct observation of the behavior has no clear standing among the tools that are designed and used to assess cognitive functions.

The experience of authors interacting with diabetics and some other patients with chronic disease indicates that the behavior observed in this group of patients is not significantly different from that of healthy ones.

Like their healthy counterparts, these patients didn’t reveal any notable dysfunction identifiable by an experienced observer in terms of personal interaction, family communications, participation in peer groups, attendance in social learning settings, and understanding social concepts.

However, the results of studies conducted by the authors of the present article and many others confirm the existence of some differences in the cognitive domains of children and adolescents with diabetes and their healthy peers in terms of memory, executive function, attention and concentration [10]. Nevertheless, the point of course is that this view is on the basis of contractual assessment tools that are designed to measure cognitive processes, while it seems that no significant difference is found taking a behavioral point of view into account, which is the only observable output of a healthy cognitive system being emphasized in heavenly teachings.

In general, it seems that a new methodology should be developed to assess the impact of diseases, with special attention to chronic diseases such as diabetes on cognitive system, not just cognitive processes. This method, which we have named it COGNITOMICS should seek to assess the individual’s cognitive system directly rather than assessing the components of cognitive processes on a quantitative basis, wherein the careful observation of behavior is the only way of this assessment. However, the important notion in Cognitomics is the principles of normal behavior definition, which may not be consistent with the behavior of the majority of the population based on religious teachings.

In this methodology, the first step is to define the principles of a good behavior, and then normal behavioral tendencies need to be illustrated accordingly.

Later on, the cultural-adopted tools required for observing and recording of these behaviors will be available to be used in the clinical assessment of patients with emphasis on real life observations.

In summary, a novel methodology is needed while assessing the impact of diseases such as diabetes on cognition based on heavenly teachings in which cognition is believed to be the main source of human bliss and not just some measurable quantitative components. This new method paves the way for an accurate assessment of a normative behavior.

The authors will develope proper tools for Cognitomics in future and will evaluate its effectiveness.
